# Geographic variation in pneumococcal vaccine efficacy estimated from dynamic modeling of epidemiological data post-PCV7

**DOI:** 10.1038/s41598-017-02955-y

**Published:** 2017-06-12

**Authors:** Erida Gjini

**Affiliations:** Instituto Gulbenkian de Ciência, Rua da Quinta Grande 6, Oeiras, Portugal

## Abstract

Although mean efficacy of multivalent pneumococcus vaccines has been intensively studied, variance in vaccine efficacy (VE) has been overlooked. Different net individual protection across settings can be driven by environmental conditions, local serotype and clonal composition, as well as by socio-demographic and genetic host factors. Understanding efficacy variation has implications for population-level effectiveness and other eco-evolutionary feedbacks. Here I show that realized VE can vary across epidemiological settings, by applying a *multi-site-one-model* approach to data post-vaccination. I analyse serotype prevalence dynamics following PCV7, in asymptomatic carriage in children attending day care in Portugal, Norway, France, Greece, Hungary and Hong-Kong. Model fitting to each dataset provides site-specific estimates for vaccine efficacy against acquisition, and pneumococcal transmission parameters. According to this model, variable serotype replacement across sites can be explained through variable PCV7 efficacy, ranging from 40% in Norway to 10% in Hong-Kong. While the details of how this effect is achieved remain to be determined, here I report three factors negatively associated with the VE readout, including initial prevalence of serotype 19F, daily mean temperature, and the Gini index. The study warrants more attention on local modulators of vaccine performance and calls for predictive frameworks within and across populations.

## Introduction

Over the last 20 years, the global epidemiological dynamics of *Streptococcus pneumoniae* have been under intense investigation. Typically carried asymptomatically in the human nasopharynx, pneumococcal bacteria also cause disease, such as otitis, pneumonia and meningitis, and are recognized as one of the most important causes of morbidity and mortality worldwide^1^. Prevention of bacterial colonization critically reduces chances of spread and transmission, thus indirectly protecting from pneumococcal disease^2^. Under this premise, since 2001, control of this pathogen has focused on host immunization with pneumococcal conjugate vaccines, via which protection applies not only to vaccinated individuals, but also to the rest of the population, a phenomenon known as herd immunity.

The first licensed multivalent pneumococcal conjugate vaccine (PCV7), confers protection against 7 of >90 pneumococcal capsular serotypes^3^, namely against 4, 6B, 9V, 14, 18C, 19F and 23F, by eliciting anti-capsular antibodies^4, 5^. This vaccine has now been followed by higher-valency vaccines, such as PCV10 and PCV13^6^. From early studies on PCV7^7–10^ the protective effect of pneumococcal vaccination became clear, in terms of reduction of colonization by vaccine serotypes (VT), as well as their associated disease. For example, results from an early vaccine trial^11^ in Gambia, West Africa, showed that vaccine serotype carriage following vaccination decreased in children who had received two doses of the vaccine (odds ratio OR 0.22 [95% CI: 0.08–0.61]), but was accompanied by an increase in non-vaccine type (NVT) carriage (OR 0.65 [95% CI: 0.29–1.42]). A later community-randomized controlled trial^12^ investigating the effect of PCV7 on nasopharyngeal colonization among American Indian infants showed that vaccinees were less likely to be colonized with VT (OR 0.40 [95% CI, 0.23–0.67]), but again were more likely to be colonized with NVT pneumococci (OR 1.67 [95% CI: 1.02–2.78]).

After mass childhood vaccination was introduced in routine immunization programs around the world, epidemiological surveys have documented the response in immunized host populations to this vaccine, e.g. in the USA^13–15^ in Switzerland^16^, England^17, 18^, the Netherlands^19^, and many other countries. These reports have described the post-vaccination changes in prevalence of pneumococcal bacteria across settings, microbial population structure, serotype and clonal distribution and antibiotic resistance patterns, elucidating the great diversity and evolutionary potential of pneumococci. While vaccine serotypes declined, overall colonization rates were seen to remain generally stable, partly due to serotype replacement, the process by which non-vaccine serotypes rose in carriage as well as disease (reviewed in ref. 20). Although variation in vaccine effects and serotype replacement across communities has been recognized^20, 21^, the reasons for this variation are unclear. As the net benefit of vaccination critically depends on the balance between vaccine protection and serotype replacement, and the complex relationship between carriage and disease, an important challenge in pneumococcus epidemiology remains to understand and predict post-vaccination dynamics, going beyond descriptive approaches^22, 23^. This includes capturing variation between settings under the same mechanistic framework of pneumococcus transmission. Monitoring disease outcomes, which are often the endpoint in clinical trials^24^, provides limited insight into the underlying mechanisms that determine herd immunity and serotype replacement^25, 26^. For this, carriage data are essential, needing new statistical approaches^27^, and dynamic modeling^28^. In this paper, I apply one such integrative approach to post-vaccination data from different settings, uncovering epidemiological parameters responsible for the differences in serotype replacement rates.

By fitting the same dynamic model to several studies reporting post-vaccine dynamics in day care centres, I address the question of what is the realized vaccine efficacy, consistent with the serotype replacement reports in different countries, and what may be the underlying local underlying factors. Although pre-licensure trials offer basic estimates of vaccine efficacy^7, 29^, they typically focus on disease outcomes in one particular population, which cannot be directly extrapolated to new untested contexts. After mass immunization takes place in another transmission setting, it is possible that a different net protection at the individual level may result. Such variation could be driven by environmental variability, local serotype and clonal composition, as well as by demographic, social or immuno/genetic factors in the host population. Accurate estimates of vaccine efficacy against serotype acquisition are crucial to subsequently predict or interpret impact on pneumococcal disease^22^.

Given a multitude of pneumococcus transmission models^30–33^, the choice of the appropriate one for retrospective vaccine assessment is not straightforward. The type and detail of the data inevitably constrain the complexity of possible model structures. A solution is to start with simple models which afford questions at a general level, and then gradually build in complexity for finer questions when feasible. The advantage of coarser-grain descriptions is that parameter readouts are likely to be more robust and comparable across settings. In this spirit, the present study uses a simple epidemiological framework^34^, based on a neutral model for pneumococcus dynamics, to integrate temporal observations pre- and post- PCV7. The model accounts for time of survey, transmission intensity and variation in coverage rates, recognized as strong sources of heterogeneity in meta-analyses^35^. Moreover, this framework provides a tool to comparatively assess vaccine performance in field settings, incorporating the basic epidemiological feedbacks, including herd immunity and serotype replacement, typically neglected in vaccine trials and in purely statistical post-licensure approaches^12, 23, 27, 36^.

The data consist of cross-sectional prevalences of pneumococcal carriage in day care centres (DCCs) in Portugal, Norway, France, Greece, Hungary and Hong-Kong, from studies conducted before and after implementation of PCV7. DCCs are an important setting of transmission of pneumococcus in developed countries. Pre-school children display highest nasopharyngeal carriage rates, and act as the main reservoir for pneumococcal spread in the community^37, 38^, consequently becoming critical vectors of herd immunity effects. It has been shown that pneumococcal transmission may take place as micro-epidemics driven by the day care centres^39^. Often the serotypes identified as dominant in some DCCs, i.e., those found to cause micro-epidemics, are not necessarily the most transmissible ones. It is thus probable that most serotypes transmit in a similar fashion in the child population, and micro-epidemic patterns and neutral micro-epidemic bacterial evolution^40^ result primarily from heterogeneous transmission between interconnected host clusters.

The analysis of pneumococcus dynamics has persistently lingered between a serotype-specific approach and neutral models based on equivalent trait approximations. The empirical evidence appears mixed: some studies support slight variation in serotype acquisition, competition and clearance rates^41–44^, others conclude that serotype differences are not significant^45, 46^, others find differences only in clearance rates and not in acquisition^47^. Furthermore, when trying to match serotype trait hierarchies across epidemiological settings^42, 43^, inconsistent estimates emerge, questioning universally fixed serotype traits. Given the inconclusive picture, and the diversity of pneumococci beyond the serotype level, multiple models have adopted neutral formulations, assuming equal transmission and clearance rate across serotypes^31, 38, 48–50^. Some of these describe pre-vaccination dynamics^50^, others predict vaccination impact across host age classes^49^, or interpret epidemiological dynamics after vaccination^31, 34^. Other models neglecting serotype differences focus on transmission fluxes between child care centers and the community^51^, pneumococcus interactions with influenza^52^, and seasonality^53^.

In this paper, I also adopt the neutral perspective to model pneumococcus colonization dynamics in young children attending day care. Serotypes are partitioned in two functionally relevant groups: aggregated vaccine and non-vaccine serotypes, and equal traits across serotypes are assumed as a first order approximation, averaging out underlying clonal diversity. In this context, serotype replacement is exclusively driven by vaccination. With a *multi-site-one-model* approach, my aim here is threefold: (i) to use minimal assumptions to tie multiple strands of evidence together, making the most of sparse data, (ii) to gain a deeper retrospective insight into vaccine efficacy of PCV7, by estimating setting-specific parameters underlying dynamic serotype replacement, (iii) to assess variability in realized vaccine protection across settings, and generate hypotheses for this variation.

## Results

### Pneumococcus colonization data from different settings

Data were extracted from 6 different cross-sectional studies on pneumococcus prevalence before and after vaccination with PCV7: the studies are summarized in Tables 1 and 2. Because not all the data published in these papers are used in the model fitting, in the interest of clarity and reproducibility of my analyses, here I summarize the key information that is useful in the context of this study. The criteria for selection of studies were: (i) focus on day care transmission setting; (ii) report on multiple time points; (iii) relatively large sample size; and (iv) no interference with more recent vaccines. Each study analyzed pneumococcus colonization in day care centres in a different country: in Oeiras, Portugal^54, 55^, in Oslo, Norway^56, 57^, in Alpes Maritimes, France^58^, in Larissa, central Greece^59^, in Szeged, Hungary^60, 61^, and in Hong Kong^62^. Out of these, only three studies reported co-colonization, respectively the Norwegian and Portuguese study, which have also been partly modelled previously^34, 50^, and the Greek study.

**Table 1:** Characteristics of the studies in daycare settings from different countries before and after PCV7 vaccination.

Country	Survey period	Mean sample size	Mean age (range) in months	Time in DCC	Vaccine coverage % (*ρ*) (mean over all ages)	Co-col. (%)	Ref.
Portugal	2001–2007	359	48 (12–71)	24	17, 32, 56, 65, 63, 75	10%	54, 55
Norway	2006–2008	599	41 (12–60)	32	10, 40	14%	56, 57
France	1999–2008	321	23 (3–39)	17	0 (99–01), 37, 37, 68.4, 68.4, 90.1	—	58
Greece	2005–2009	662	47 (13–76)	27	12.9, 32.6, 70.1, 95.5	3.8%	59
Hungary	2001–2009	413	54 (36–72)	18	0, 10 (02–05), 21.5, 38.5, 38.5	—	60, 61
Hong Kong	2000–2010	2094	49 (24–72)	24	0 (00–05), 20.4, 26.7, 35.7, 43, 43	—	62

Mean age of children in each study and expected duration of DCC attendance (see S2.2 for details) are given in months. Vaccination proportions per year were extracted from the children that that received at least one dose of PCV7 across the studies. When information on vaccination coverage for intermediate years was missing, the same value as in the preceding year was assumed.

**Table 2:** Pneumococcus prevalence data from daycare settings in different countries.

Country	Sampling months: (*t*)	Number of carriers: *n*_*V*_(*t*) + *n*_*N*_(*t*)	Carriers of PCV7 serotypes: *n*_*V*_(*t*)	Sample sizes: *n*(*t*)
Portugal	0, 72	173, 319	102, 50	270, 449
Norway	0, 24	470, 474	212, 97	606, 592
France	0, 36, 60, 84, 108	161, 172, 182, 168, 155	123, 128, 89, 35, 6	298, 294, 334, 335, 343
Greece	0, 12, 24, 48	370, 206, 286, 334	132, 88, 33, 33	769, 494, 566, 820
Hungary	0, 78, 112	34, 38, 357	19, 17, 46	95, 121, 1022
Hong Kong	0, 120	383, 347	253, 190	1978, 2211

For Portugal and Norway more data were available and were used than shown here (see Methods 4).

All studies used sensitive serotyping techniques on nasopharyngeal swabs collected from healthy children attending day-care. The settings varied in serotype and clonal composition, vaccination coverage and time of follow-up (Table 1). The study sample sizes varied between a minimum total of 240 children a maximum of 2094 children in Portugal and Hong-Kong respectively. Among other findings, all studies reported decreasing trends in the proportion of colonized hosts with serotypes included in PCV7 after implementation of vaccination. All studies reported no temporal trend with regards to overall colonization prevalence, except the Hong Kong and the French study, which showed slightly declining carriage over time.

The number of circulating serotypes across time periods was between 26 and 33 in all studies. The four most prevalent serotypes before vaccination in each country varied slightly in their ranking: 19F, 23F, 11A/D, 6B (Portugal), 6B, 19F, 15B/C, 23F, (Norway); 6B, 19F, 23F, 14 (France); 6A, 11A, 10A, 35F (Greece); 14, 19F, 23F, 6A/B (Hungary); 6B, 19F, 23F, 14 (Hong-Kong) (see Table S1 for details). The Portuguese and Norwegian study reported in addition specific colonization prevalences with PCV7 and non-PCV7 serotypes both in single and multiple carriage after vaccination. The number of time points per study is 2 (Portugal), 2 (Norway), 5 (France), 4 (Greece), 2 (Hong Kong) and 3 (Hungary). The exact number of day care centres (DCCs) varied slightly: from 11–12 DCCs in Portugal, to 27–29 in Norway, 25 in France and 21 in Greece. For model fitting, aggregated colonization data across DCCs are used, assuming homogeneous mixing between all the children. To facilitate comparison across settings, host age structure is neglected, approximating children attending day care, aged between 1–5 years as a compact age group with similar characteristics, spending on average about 2 years in the DCC environment (see S2.2 for details).

### Fitted transmission model

**Figure 1:**
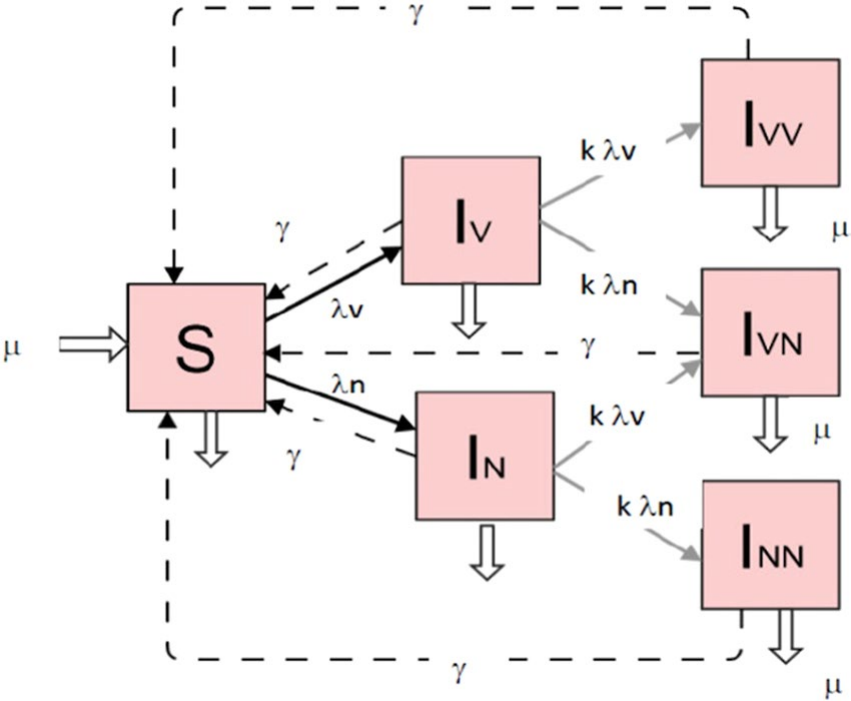
Model flow diagram in the absence of vaccination. S, susceptible, *I*_*V*_, single carriers of any vaccine serotype, *I*_*N*_, single carriers of any non-vaccine serotyoe, *I*_*VV*_ double carriers of any two vaccine serotypes, *I*_*NN*_, double carriers of any two non-vaccine serotypes, *I*_*VN*_, double carriers of a vaccine and non-vaccine serotype. With vaccination, the structure of the model is doubled to account for vaccination status of each epidemiological class. The parameters are defined in the text. See Methods 1–3 for details, and Supplementary Material S1 for full ODE equation formulation.

The dynamics of pneumococcal colonization and co-colonization among young children attending day care are modelled with a previously published neutral model^34^. The susceptible-infected-susceptible (SIS) formulation tracks six epidemiological compartments, including susceptible hosts, *S*, hosts colonized by any one vaccine serotype (here PCV7) *I*_*V*_, hosts colonized by one non-vaccine serotype, *I*_*N*_, and co-colonized hosts *I*_*VV*_, *I*_*NN*_, and *I*_*VN*_ with two vaccine serotypes, two non-vaccine serotypes or one of either group, respectively (Fig. 1). With vaccination, the model equations double, to account for vaccination status of each epidemiological class. A fraction *ρ* of all new susceptible hosts, recruited at rate *μ*, is vaccinated, a quantity that can vary across geographical settings and also from year to year (Table 1). Vaccinated hosts display partially reduced susceptibility to VT, relative to non-vaccinated ones, given by the scaling parameter 1 − *VE*, where vaccine efficacy against VT, denoted as *VE* (0 ≤ *VE* ≤ 1), describes the net per-capita protection aggregated over all serotypes included in the vaccine. Other studies in the literature^31, 33^, also represent aggregated vaccine efficacy with a single parameter, assuming similar and long-lasting protection on average across all PCV7 serotypes. Further details on model structure, features and assumptions are given in Methods 1–3 and Supplementary Material S1.

In line with previous neutrality assumptions^31, 38, 48, 50^, also here, phenotypic equivalence across serotypes, with respect to basic colonization capacity (transmission rate *β*), clearance rate (*γ*) and direct competition coefficient in co-colonization (*k*) is assumed. The pneumococcus force of infection for each serotype group (λ_v_ and λ_n_) depends explicitly on their prevalence in the population, including contribution by single and double carriers. This feature is critically reflected in the model initial conditions, when calibrated to each dataset under the assumption of pre-vaccine endemic equilibrium (see Methods 2). In all settings, initial conditions are adjusted according to local serotype prevalences, linking the ensuing dynamics to reported VT/NVT distribution before vaccination. Once initialized, the vaccination model trajectories are fitted to the remaining cross-sectional observations, specific to each study (see Methods 4 and data in Table 2). Stochasticity due to sampling effects is accounted for using a multinomial distribution with a different sample size, for the likelihood of the data at each time point. Parameter estimation is performed in a Bayesian framework in MATLAB, using adaptive Metropolis-Hastings MCMC^63^.

### Estimated basic reproduction number *R*_0_ and serotype competition parameter *k*

An endemic persistence equilibrium requires *β* > *γ* + *μ*, corresponding to the classical threshold for the basic reproduction number^64^: ${R}_{0}=\frac{\beta }{\gamma +\mu } > 1$. Provided that *R*_0_ > 1, the pneumococcus endemic colonization state is asymptotically stable. Because it is a measure of transmission intensity, the higher *R*_0_ is, the higher the endemic prevalence of the pathogen, and the higher also the co-colonization. In particular, susceptibles, single colonization and co-colonization prevalences are given by:

1 $${S}^{\ast }=\mathrm{1/}{R}_{0},\quad {I}_{1}^{\ast }={I}_{V}^{\ast }+{I}_{N}^{\ast }=\frac{1-\mathrm{1/}{R}_{0}}{1+k({R}_{0}-\mathrm{1)}},\quad {\rm{and}}\quad {I}_{2}^{\ast }={I}_{VV}^{\ast }+{I}_{NN}^{\ast }+{I}_{VN}^{\ast }=k({R}_{0}-\mathrm{1)}{I}_{1}^{\ast }\mathrm{.}$$

Serotype distribution at endemic equilibrium under neutrality is thus flexible regarding single and multiple carriage combinations, but globally satisfies ${P}_{V}^{\ast }+{P}_{N}^{\ast }=1-\mathrm{1/}{R}_{0},$ relative to VT and NVT prevalences, a conserved relationship even after vaccination. At a global level, in a symmetric multi-type system, if transmission rate *β* (or analogously *R*_0_) stays constant over time, targeted vaccination is not expected to alter the overall magnitude of single and co-colonization; only shift the serotype composition towards non-vaccine types, a picture supported by serotype replacement patterns post-PCV7 across countries^13, 16, 17^, and the majority of our data. The basic reproduction number in each setting was assumed constant over time, and estimated within the integrated fitting procedure for the entire time-series.

**Figure 2:**
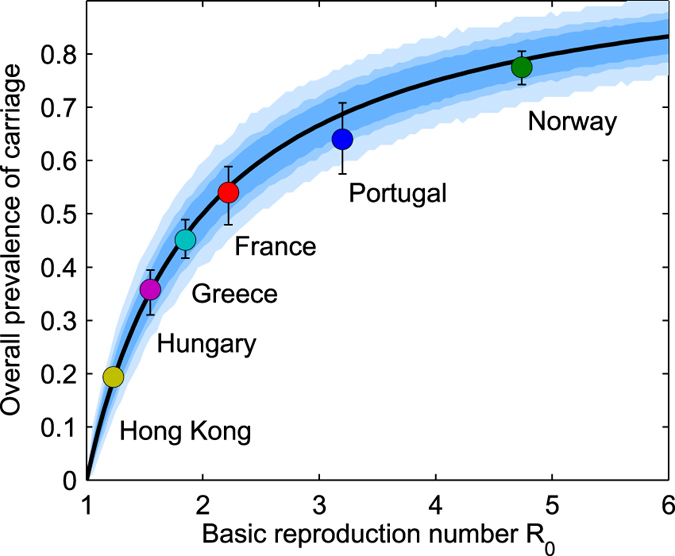
Transmission intensity vs. overall pathogen prevalence in a SIS framework. The mean *R*_0_’s estimated from our datasets are matched with the expected overall prevalence of pneumococcal carriage in the pre-vaccine era. The bars reflect the 95% confidence interval for binomial proportions over 300 simulations with same *R*_0_, taking into account the mean sample size in each setting (Table 1). The line depicts the theoretical nonlinear relationship expected by the model (Eq. (1)). The shaded regions represent the 95% quantile of simulations under different sample sizes: *n* = 100 (light blue), *n* = 250 (darker blue) and *n* = 500 (darkest shade).

The values for *R*_0_ estimated for these studies, range between 1.2 and 4.7 (Table 3), indicating variable transmission intensity within day-care attendees across geographical sites, lowest in Hong Kong and highest in Norway, respectively (see Fig. 2). Each child carrying pneumococcus within the DCC causes thus on average 2.5 new colonizations over his entire carriage period. The model estimates also a high competition between serotypes acting at acquisition, in the range of 90–95% inhibition of co-colonization, independently from the datasets that reported co-colonization, namely the Norwegian, Portuguese and Greek settings.

**Table 3:** Parameter estimates for pneumococcus transmission and PCV7 vaccine efficacy (*VE*) in day care settings.

Country	Basic reproduction number *R*_0_	Competition parameter *k*	Vaccine efficacy *VE*	Transmission rate *β* (month^−1^)
Portugal	3.18 (2.87, 3.53)	0.08 (0.06, 0.11)	0.11 (0.09, 0.14)	2.36 (2.13, 2.62)
Norway	4.77 (4.28, 5.30)	0.04 (0.03, 0.05)	0.43 (0.35, 0.50)	3.49 (3.13, 3.88)
France	2.22 (2.14, 2.31)	—	0.19 (0.16, 0.21)	1.68 (1.62, 1.74)
Greece	1.85 (1.79, 1.91)	0.045 (0.04, 0.05)	0.21 (0.17, 0.25)	1.36 (1.32, 1.41)
Hungary	1.55 (1.49, 1.62)	—	0.27 (0.20, 0.31)	1.17 (1.12, 1.22)
Hong Kong	1.23 (1.22, 1.24)	—	0.08 (0.05, 0.10)	0.91 (0.90, 0.92)

The means and the 95% credible intervals for each parameter are obtained from the posterior distributions. The competition coefficient *k* is estimated from co-colonization data in some cases, and set to a fixed value in others (—), equal to 0.05, a criterion used to calibrate model initial conditions (see Methods 2). The basic reproduction number is derived as: ${R}_{0}(i)=\frac{\beta (i)}{\gamma +\mu (i)}$. Setting-specific birth/death rate *μ* for the SIS model was assumed as the inverse of mean duration of DCC attendance for children in each study (see Table S2 for results based on 1/mean age instead).

### Links with other studies

The *R*_0_ values resemble *R*_0_ estimates for other respiratory pathogens such as influenza^65^. The highest *R*_0_ was estimated for Norway, displaying highest carriage prevalence. This may be related to the low rate of antibiotic use and high rate of DCC attendance in Norway compared to other settings^56^. Understanding *R*_0_ variation is important when forecasting population-level impact of a particular vaccination programme^66^. For example, when contemplating vaccines based on serotype-transcending antigens, depending on the value of *R*_0_ in each setting, a different vaccination coverage with such universal vaccine would be needed to achieve the same impact. In addition, transmission intensity critically shapes the pathogen evolutionary potential, by affecting nonlinearly prevalence of carriage (Fig. 2) and multiple-carriage, the latter being key in bacterial recombination^67, 68^.

Competition coefficient estimates are consistent with earlier studies from Denmark and England^31, 69^. In fact, the pre-PCV7 Norwegian data from 2006 has also been analyzed previously^50^, with a stochastic SIS Markov model for transmission within DCCs. Strikingly, with the simplified deterministic model in this paper, applied to both pre-and post-vaccination data, and neglecting infection hazard from the community, very similar estimates emerge for both transmission rate *β* (3.59, 95% CI: 2.8, 4.6) within DCC and competition between serotypes at acquisition *k* (0.1, 95% CI: 0.06, 0.14), suggesting a minor influence of stochastic effects on SIS model parameter estimation^70^, at least at this particular sample size (*n* ≈ 600). For the other studies not reporting co-colonization, *k* could not be estimated from the data. Instead, a fixed value for *k*(=0.05) was used to simulate their trajectories, in line also with earlier estimates for this parameter^31, 50, 69^.

The resulting monthly child-child transmission rate *β* for France in this study, around 1.68 (95% CI: 1.62, 1.74), was similar, although somewhat higher, to previous estimates obtained from a longitudinal French modeling study^45^ with schoolchildren 3–6 years in 2000 (1.45, 95% CI: 1.30, 1.59). The difference could partly be explained by the fact that the children in the Alpes-Maritimes study^58^, modelled here, represent a younger age group, more prone to colonization, and by the fact that the present epidemiological model neglects transmission from the non-DCC community, thus attributing all the force of infection to the DCC environment. Interestingly, the *β* estimated for Greece 1.36 (95% CI: 1.32, 1.41) compares better with the range of the French study^45^, probably because the children age ranges match more closely. The lowest transmission rate estimated for the Hong-Kong day care setting, seems closer to the transmission rates within families in Bangladesh, between 0.64 and 0.84 per month estimated previously^46^, while transmission rates for Hungary are consistent with another French study reporting within DCC transmission rates between 1.04 and 1.18^42^.

### Vaccine efficacy against acquisition of PCV7 serotypes

**Figure 3:**
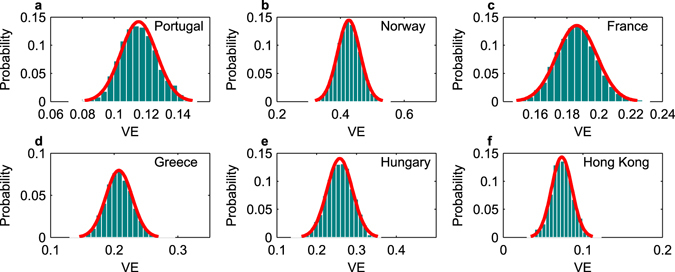
Posterior distributions for heptavalent vaccine efficacy against PCV7 serotypes, estimated in a setting-specific manner. Parameter inference is based on dynamic model fitting to cross-sectional data in day care settings from different countries, accounting for different transmission intensities, initial serotype distribution, and vaccine coverage. Red lines indicate normal distribution fits. Numerical results are detailed in Table 3.

**Figure 4:**
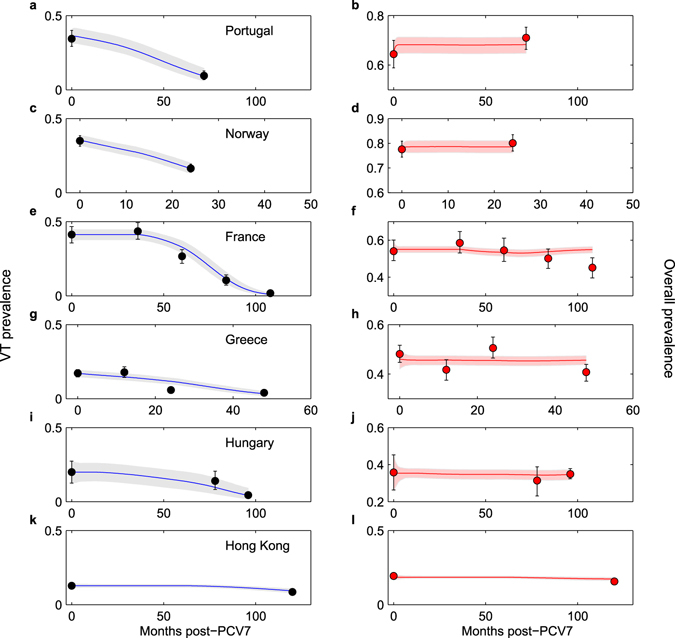
Observed and fitted prevalence of asymptomatic carriage of pneumococcus in day care centres by country. The model-predicted 95% credible envelopes for temporal trajectories of VT prevalence (left panels) and overall prevalence (right panels) after vaccination are superimposed on observed data in different day care settings at specific time-points. (**a**,**b**) Portugal. (**c**,**d**) Norway. (**e**,**f**) France. (**g**,**h**) Greece. (**i**,**j**) Hungary. (**k**,**l**) Hong Kong. The credible envelopes were computed using 500 simulations with different parameter combinations sampled from the joint posterior, and here account only for the uncertainty around parameter values and initial conditions. For the credible envelopes accounting for sampling uncertainty throughout time (with mean sample size as in Table 1) see Fig. S6.

The other parameter estimated with this model is vaccine efficacy against acquisition of PCV7 serotypes, aggregated over all VT (Fig. 3). This parameter reflects the reduced susceptibility in the acquisition of VT, either as primary or secondary colonizing serotypes, in vaccinated relative to non-vaccinated individuals. The 6 independent estimates for each dataset, obtained by fitting the dynamic model to post-vaccine observations, accounted for differences in vaccination coverage (Fig. S1), time of survey, and transmission intensity between settings. As summarized in Table 3, the reported values of *VE* suggest differential relative vaccine protection, experienced on average by each vaccinated child in the age-groups considered, across settings. According to this model, the four datasets from Portugal, France, Greece and Hong Kong are consistent with lower values for vaccine efficacy, around 10–20%, while the Norwegian and Hungarian data are consistent with a higher value of vaccine efficacy around 30–40% (see Fig. 3), with the 95% credible interval for Norway VE in the range 30–50%. Notice that the model can distinguish between differences in efficacies that are invariant to serotype distribution prior to vaccination, because the initial conditions for each study are calibrated to reported local serotype frequencies aggregated as VT and NVT prevalences, at the first time point (see Methods 2). The predicted trajectories for the epidemiological dynamics with the estimated parameters, drawn from the posteriors (Table 3), are shown in Fig. 4, matching well the pneumococcus prevalence observations in each country.

### Sensitivity to model assumptions

The estimated VE values remain robust to eventual changes in clearance rate *γ* across settings (affecting only *R*_0_), and to the value of serotype competition parameter *k*, affecting mainly the ratio between dual and single serotype carriage. However, they are slightly more sensitive to the assumed *μ* (susceptible host recruitment rate), which besides modulating *R*_0_, multiplies directly the vaccination coverage *ρ*. When *μ* increases, turnover rate in the modeled population increases, and vaccine efficacy estimates decrease slightly for the same coverage (Fig. S2). In the case of using 1/mean age for *μ* (Table 1), as an alternative to 1/mean duration of DCC attendance, the estimated vaccine efficacies varied slightly, with Norway and Hungary still displaying highest values around 45%, and *R*_0_ values kept unchanged (see Table S2 for results).

When using a continuous vaccination assumption instead of a step-wise increasing coverage, i.e. holding fixed the vaccination coverage, throughout the years of each survey, the sensitivity analysis for the estimated VE indices yielded somewhat lower values in absolute terms to the above ones reported here, yet preserving the rank-ordering between multiple sites, and showing little dependence on the assumed *μ* (Figs S3 and S4). When vaccination coverage is held constant, e.g. around the median of values in Table 1, the resulting VE estimates display an overall downward bias, because in this case, the model over-estimates the magnitude and sequential effects of the positive feedbacks that arise in the early stages of vaccination, due to a higher-than-realistic assumed coverage in year 1. As vaccine coverage *ρ* and vaccine efficacy *VE* can be traded-off against one another, potentially yielding indistinguishable model trajectories, it is important to realize that VE estimates given a certain dataset remain coupled to the originally assumed coverage rates.

While here the model-based vaccine efficacy estimates assume stable endemic transmission, in reality there may be cases where prevalence has declined. In particular the French dataset indicated a slight temporal trend for reduction in overall prevalence of pneumococcus, which suggests a time-varying *R*_0_ for this country, probably driven by other interventions than vaccination (e.g. change in antibiotic use). Although, these changes were not addressed in the model-fitting, in Fig. S5, model-predicted serotype replacement dynamics for different values of vaccine efficacy and lower transmission are illustrated, and the difference appears minimal. Under a neutral model, such as the one applied here, changes in *R*_0_ should not impact significantly on the relative rate of decline of targeted serotypes post-vaccination. By contrast, under a non-neutral model, for example where direct competition at co-colonization may be asymmetric, the magnitude of transmission intensity, *R*_0_, is expected to influence the hierarchical dynamics between types both in the absence^71^, and presence of vaccination^72^.

### Possible factors of vaccine efficacy variability across settings

In spite of all simplifying assumptions, model estimates of vaccine efficacy for PCV7 emerging from this study are close to existing estimates in the literature^31, 73^. For example, Choi *et al*.^31^, using a similar framework, estimated vaccine efficacy in England *VE*_*c*_ = 50%, close to the value for Norway estimated here. More recently, a meta-regression study^73^, pooling data from different settings together, also reports that the aggregate *VE*_*c*_ for all PCV7 serotypes 6 months after completion of the vaccination schedule around 57% (95% CI: 50, 65%), tending to an aggregate *VE*_*c*_ of 42% (95% CI: 19, 54%) at 5 years. Compared to these earlier approaches, a novel effect emerging from the present study, calling attention, is the heterogeneity in vaccine efficacy against PCV7 serotypes across geographical settings, which requires a deeper look at the data and our model formulation. Among many possible factors, here I explore only three in a statistical sense, inviting further examination of the local modulators of vaccine performance in the field.

### Serotype-specific efficacies and aggregation effects

**Figure 5:**
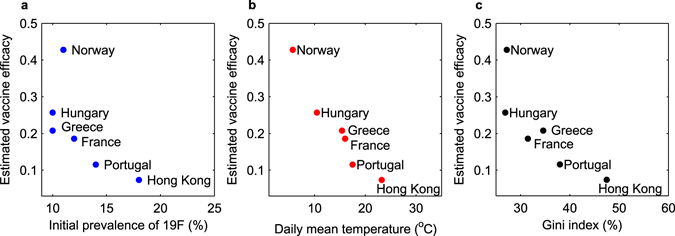
Possible factors for variation in vaccine efficacy against acquisition. (**a**) Prevalence of serotype 19F pre-PCV7 and model-based vaccine efficacy estimates. The decreasing trend suggests that high initial frequency of a serotype for which vaccine protection may be inferior (e.g. 19F) could influence model estimates of the net realized vaccine efficacy against PCV7 serotypes when aggregated together, (*R* ^2^ = 47%, (*R* ^2^-adj = 11%, GLM fit: *ρ* = −0.0003, *p* = 0.10 n.s.). (**b**) Temperature across countries correlates with vaccine efficacy values estimated by the model (*R* ^2^ = 92%, *R* ^2^-adj = 87%, GLM fit: *ρ* = −0.019, *p* < 0.005 significant), suggesting a possible environmental factor in immunomodulation and vaccination responses. (**c**) Income inequality, as a proxy for transmission heterogeneity, is associated negatively to realized vaccine efficacy extracted by the model (*R* ^2^ = 68%, *R* ^2^-adj = 46%, GLM fit: *ρ* = −0.013, *p* < 0.05 significant). More details are provided in the Supplementary Tables S4 and S5. The fitted regression lines are displayed in Fig. S7.

The first is related to a main assumption of the aggregated (VT/NVT) model, namely that vaccine efficacy is equal against all 7 serotypes included in the vaccine, which although previously assumed, is clearly an approximation. Lower vaccine protection against acquisition of some PCV7 serotypes, for example against 19F, has been observed in some studies^74, 75^, and confirmed by more recent meta-analyses^73^. Such observation implies that vaccine type composition prior to vaccination, in terms of individual frequencies in each country, could be important, besides their aggregated prevalence as a group. Since the dynamic model does not describe single serotypes and serotype-specific efficacies, aggregation effects may introduce bias in overall vaccine efficacy estimation. Indeed, when inspecting the VT composition in each setting and explicitly considering the frequency of serotype 19F prior to vaccination, it emerged that in those settings where 19F was initially more prevalent, model fitting led to a lower apparent vaccine efficacy against PCV7 serotypes (Fig. 5a), suggesting a role for the serotype aggregation effect. Although this trend did not reach statistical significance with these datasets, it is not surprising, given that serotype-specific vaccine protection and serotype-specific protection waning rates have been reported for PCV7^73^.

### Environmental gradients: the role of temperature

A second potential source of modulation of vaccine efficacy locally could be environmental factors, such as temperature, humidity, ultraviolet (UV) radiation, typically varying with geographical latitude. It has been suggested that exposure to UV radiation, as in sunlight, can modulate immune responses in animals and humans^76^. This immunomodulation can sometimes be deleterious, as documented by experimental animal studies where UV exposure was shown to impair resistance to many infectious agents, including bacteria, viruses, and fungi^77–79^. When testing among our results for an association between setting-specific temperatures (daily mean, averaged over the year, (see Table S4)) and the model-estimated pneumococcus vaccine efficacies, a significant association emerged, whereby in cooler climates, PCV7 vaccine protection was realized at higher levels (see Fig. 5b). As temperature correlates with latitude and UV exposure, it is possible that environmentally-induced immunomodulation across geographical settings could interfere with the net individual protection against PCV7 serotypes conferred by the vaccine. Naturally, this hypothesis warrants further studies across more settings and experimental evidence for substantiation. Yet, geographical effects and gradients in auto-immunity have been established, indicating decreasing auto-immune disease along the north-south axis^80^. Another strand of evidence has implicated temperature with many growth and virulence properties in bacteria^81^. In the particular case of pneumococci as respiratory tract colonizers, thermoregulation across mucosal surfaces might contribute to modulating their biological interaction with vaccine-induced anti-capsular antibodies.

### Transmission heterogeneities in the host population

Third, the model employed here assumes a homogeneous underlying population with regards to pneumococcus transmission and focuses only on the day care setting. It is known that heterogeneity within host populations is very important for transmission dynamics of infectious diseases and for the design of control policies. A statistical pattern known as the 20/80 rule has been shown to apply to a variety of disease systems^82^, implying that control programs targeting the core 20% group of transmission are potentially highly effective in reducing levels of infection in the population as a whole, compared to programs that fail to reach all of this group. Mathematical models have shown that the impact of control measures such as vaccination depend crucially on the magnitude of heterogeneities in disease risk, and is lower when these heterogeneities are higher^83^. It is not unreasonable to assume that such transmission heterogeneities may be interwoven with economic disparities in a given population. Under this premise, using the Gini coefficient, a measure of income inequality^84^, as a proxy for transmission heterogeneity, where a Gini coefficient of zero implies perfect equality in distribution of wealth, and a Gini coefficient of 1 (or 100%) expresses maximal inequality, I tested whether there was an association between the setting-specific Gini indices (Table S5) and the realized apparent PCV7 vaccine efficacy in each country, extracted by the model for the 6 pneumococcus DCC datasets. As shown in Fig. 5c, there was a significant negative association, where higher vaccine efficacy was found in more homogeneous countries like Norway and Hungary, and lower efficacy per individual was realized in more heterogeneous settings like Portugal and Hong Kong. This role of heterogeneity on vaccine efficacy estimation has also been predicted by earlier theoretical arguments in the context of malaria and vaccine efficacy trials^85^. Such a relationship hints at a new possible predictor of PCV impact on pneumococcal carriage and disease, besides the ones already known^23^, namely, heterogeneity in the underlying host population, which may turn out extremely relevant when interpreting reports of vaccination effects in low- and middle-income countries, starting only recently to become available.

These findings point to a clear need for a precise mathematical understanding of post-vaccine dynamics variation across settings. Even though with the current model we cannot fully resolve the underlying causes of the variation in these 6 datasets, the study calls attention on an important aspect of pneumococcus vaccination dynamics, previously overlooked: namely, the host-pathogen-vaccine interaction across a gradient of host populations. While the details of how this variable vaccine effect is achieved remain to be addressed in follow-up studies of a larger scope, variation in itself may have important consequences for site-specific responses to current and future pneumococcus vaccines, and for other interventions.

## Discussion

The effect of vaccines in immunization programmes is often described by pooling data from different populations together^21, 73^. In this study, I have taken an alternative route, by applying a unified modelling framework in parallel to pneumococcus datasets from different countries. Interpolation of colonization data before and after vaccination via dynamic model fitting provides quantitative insight on the mechanistic coupling between serotype competition for colonization, transmission intensity, and vaccine efficacy in a setting-specific manner. This allows a deeper interpretation of serotype replacement dynamics, and serves as a first step for comparative analyses. Similar *multi-country-one-model* approaches have been insightful in other epidemiological systems, for example when investigating tuberculosis transmission^86^ and malaria dynamics^87^. In our case, emergent heterogeneities in individual direct protection, after factoring out differences in vaccination coverage and initial serotype distribution across sites, raise interesting questions.

### New insights from multi-site-one-model approaches

A novelty of the present study is the challenge to the notion that vaccine protection can be described by a single number or point estimate, equal across different populations and geographic settings. The difficulties of conducting pre-licensure vaccine trials in each setting should not preclude efforts to interpret retrospectively post-vaccine dynamics in a context-specific manner. There may be multiple factors that can affect the local performance of a vaccine, and mechanistic approaches, applied retrospectively to well-sampled, detailed, cross-sectional data, can help to disentangle these, addressing not only pathogen-intrinsic variability, as typically done, but also variability of host populations. In this study, focusing on the first pneumococcus conjugate vaccine, PCV7, and fitting the same transmission model individually to six observational datasets, describing daycare colonization patterns in different countries, I uncover variable estimates for vaccine efficacy across settings. Some of these, e.g. VE in the range 30–40% (Hungary and Norway), are close to existing estimates from PCV7 vaccine trial analysis^27^ reporting VE against acquisition around 42%, and models of cross-sectional data from England^31^, reporting a vaccine efficacy against acquisition of 50%. However, the other datasets analyzed here suggest also that a lower realized vaccine efficacy, around 12–20%, may apply in countries like Portugal, France and Greece, closer to the lower end of the range (95% CI: 0.24, 0.56), estimated with previous statistical approaches using carriage as an endpoint^27^.

Recognizing its multi-factorial nature as a parameter, realized vaccine efficacy in these studies could be statistically related to three plausible factors, given the resolution afforded by the present datasets and model formulation. The first regards vaccine protection assumed uniform for all serotypes included in the vaccine, and described by a single parameter. When accounting for the fact that vaccine-induced immunity against serotype 19F is weaker than that against other PCV7 serotypes^74, 75^, a negative trend became apparent between variable initial prevalence of 19F (the difficult serotype) at the beginning of the study period, and the resulting variable apparent protection against PCV7 serotypes when pooled together. Secondly, a stronger and significant negative relationship emerged when considering temperature and variability in PCV7 efficacy across countries, warranting further studies about environmental factors in immune responses to pneumococcal conjugate vaccines. The third association related negatively a demographic factor across populations, namely the Gini indices, a measure of income inequality and plausible proxy for transmission heterogeneity, with the realized vaccine efficacy. These results have to be taken with caution as the power to detect significant correlations is realistically limited with only 6 datasets considered here. While correlation does not imply causation, many avenues for future investigation of mechanisms remain.

### The pneumococcus diversity challenge

Although pneumococcal serotypes are identified by the same polysaccharide capsule code across geographical settings, the clones corresponding to those serotypes can be vastly different, displaying potentially different growth, competition and antibiotic resistance phenotypes. For example, the proportion of isolates resistant to erythromycin was 77% in the pre-vaccination period in Hong Kong^62^, 70% in France^58^, but only 7.6% in Greece^59^, and even lower, 5.9%, in Norway^57^. While not all aspects of clonal diversity can be depicted in a practical mathematical model, it is possible that different circulating genotypes across geographical settings, by creating a unique ecological niche, can lead to a different net vaccine protection at the individual level, when serotype or serotype group is chosen as the common denominator. Crucially, antibiotic resistance phenotypes might need to be modelled in combination with serotype diversity to fully capture vaccination effects, as theoretically suggested^88^. In fact, the local basic reproduction number *R*_0_ is likely to be also a function of antibiotic use, antibiotic resistance patterns, and the local pool of circulating pneumococci in each setting.

Back to a serotype-specific view, the fact that the dominant non-vaccine serotypes vary across sites, and likely display different interaction with vaccine serotypes, could also affect the model readout of vaccine efficacy^72^. The present formulation treats NVT as the same entity in all studies, while in reality the majority target and non-target serotypes might compete differently in each case. One could therefore expect that when the NVT serotype pool is more similar across sites, then the vaccine efficacy estimates should be more similar. Without enough unequivocal evidence to inform serotype-specific parameters *a-priori*, one can try to evaluate this grouping effect *a-posteriori*, inspecting the aggregated model results, and a simple comparative test afforded by the data, supported this expectation (see Fig. S8). Estimating asymmetric VT-NVT competition across settings requires 3 additional parameters in the co-colonization model, as exemplified previously for the Portuguese data^34^. Unfortunately, such model structures, applicable to all settings in a standard manner, need better temporal and especially co-colonization resolution, unfeasible with the current data. Pneumococcus ecological niche similarity and vaccine efficacy similarity across sites could be studied in various ways beyond simply serotype comparisons.

Inevitably all models, whether mathematical or experimental, are a simplification of reality, and serotype symmetry is clearly unrealistic for polymorphic pneumococci. Yet, here we see that the neutral model can reproduce efficiently the post-vaccine cross-sectional observations, despite omitting some biological realism; an indication that slight serotype differences in life-history traits do not radically alter global dynamics^34^. Reassuringly, this model follows theoretical arguments^89, 90^, in favour of an unbiased formulation, incorporating same strain co-infection, here at the group level VT-VT and NVT-NVT, leading to a pyramid structure, in particular also advocated for pneumococcal dynamics^31^. The importance of neutral null models, which avoid mechanisms of stable co-existence for indistinguishable strains, has been previously highlighted^48^. Thus, although seemingly a strong assumption, symmetry across VT and NVT often features in earlier models^31, 38, 48, 50^, and is in principle not limited to 2 serotype groupings. Besides its analytical advantages, the symmetry approximation allows a standardized application to temporal observations in different host populations; one step closer to their integration under a unified mechanistic framework. The definition of vaccine efficacy is also more directly interpretable in such a transmission model, reflecting the relative measure of reduced susceptibility to VT acquisition per vaccinated individual, when compared to a non-vaccinated one (in this case, in a day care setting). Despite it perhaps being a slightly biased metric, due to the unrealistic symmetry assumption, it will most likely be precise and informative, thanks to the simple model structure, enabling reproducible comparison across settings and a useful start for further exploration.

### The host diversity challenge

Differences in realized net protection per vaccinated individual across settings are not unlikely. They may result from variable vaccine-induced immunity in different host backgrounds, where genetic factors of immunosuppression^91^, or local environmental factors such as nasopharyngeal microbiota (for respiratory pathogens)^92, 93^, might play a role. Different implementations of the immunisation programme can also be a source of heterogeneity, e.g. the effects of vaccination schedule, age of vaccination^94^, or prior pathogen exposure^95^. In addition, populations realistically vary along global transmission-modulating factors such as temperature, crowding, DCC attendance, age distribution, antibiotic use, and climatic conditions. Child nutritional status and interference between concurrent vaccines might also be important. All of these population-level processes, by way of interacting and often hidden nonlinear feedbacks, may exert top-down influences on the individual-level parameter readout (per-capita protection per vaccinated individual: VE), obtained when relatively simpler models are applied to data.

Revisiting the heterogeneity argument, it is important to note that a critical aspect of a dynamic model for vaccine assessment is the natural incorporation of herd immunity effects, whereby unvaccinated individuals receive indirect protection, through the immediate lower prevalence of target serotypes in vaccinated individuals. Given the data, the present model accounts for herd immunity only within the DCC environment, neglecting transmission with the outside community. Although its contribution to epidemiological DCC dynamics is not expected to be very big^50, 69, 96^, with a rate of 0.5–0.6 per month, this might vary across settings^51^. In addition, herd immunity effects outside the DCC reservoir could affect the pace of serotype replacement observed within the DCC post-vaccination. For example, higher levels of DCC attendance in a population, coupled with high vaccination coverage in this reservoir, and a certain mixing between DCC attendees and the rest of the population could eventually speed up serotype replacement, by creating more positive feedbacks in host clusters^97^.

Thus, variable DCC attendance among children across different geographical areas, might scale differently the importance of this epidemiological setting in the spread of pneumococci, and modulate differently the net herd immunity effects that feed back into the DCC environment. Such effects would necessarily shape the serotype dynamics ‘seen’ by our model. Hence, what emerges as variability in vaccine efficacy estimates, from modeling exclusively the DCC compartment, could in reality hint at different coupling, between one part and the rest of a structured population^66^. Complemented datasets including DCCs, and samples from the children’s families or rest of the community, as well as from other relevant reservoirs of transmission of pneumococci, would be necessary to properly account for these heterogeneities in a dynamic model for vaccine assessment. For wider age ranges and age-stratified colonization data, matched across studies, model extensions could also incorporate temporal heterogeneities, e.g. waning of vaccine protection, by adding explicit host age structure to the epidemiological dynamics.

### Next-generation analyses for next-generation vaccines

Considering all the epidemiological, demographic, and environmental factors underlying pneumococcus vaccine efficacy variation falls beyond the scope of this paper, and we cannot rule out any explanation with the present datasets. While the details of how this effect is achieved remain to be determined in the future, variable efficacies have been observed and studied in other pathogen systems with vaccination, e.g. in *Mycobacterium tuberculosis* and the BCG vaccine^98^. The aim of the present study is to illustrate a new way of looking at pneumococcus vaccine protection realized in the field, by adopting a setting-specific lens of analysis under a unified dynamic model. Such retrospective frameworks post-licensure could offer an additional tool to identify and quantify first-order differences in vaccine efficacy across host populations, and then lead to detailed and systematic exploration of second-order causal links and data-driven model extensions. Once the dominant sources of variability across communities are uncovered, it would be interesting to test whether they apply also to other, more recent vaccines.

Next-generation vaccines for protection against bacterial pathogens remain an area of active research^6^, for which an accurate interpretation and forecast of intervention outcomes are needed. Whenever possible, their efficacy should be evaluated in a context-specific manner, and re-assessed via multiple approaches and available data. Linking pre-licensure vaccine studies with follow-up observational surveys, and standardized dynamic models, will yield a better understanding of the underlying mechanisms of vaccine protection, their sources of variability, and downstream effects on population-level dynamics. Site-specific and comparative analyses should not be seen as questioning the importance or public health benefit of vaccination programs, but rather as a way to learn from the differences between settings^99^, and to use this understanding for better disease control and empowered vaccines globally.

## Methods

### Pneumococcus transmission model pre-vaccine

The dynamics of pneumococcus colonization and co-colonization in young children is described through a susceptible-infected-susceptible (SIS) epidemiological model, tracking the proportion of: susceptible hosts, *S*, hosts colonized by vaccine serotypes *I*_*V*_, hosts colonized by non-vaccine serotypes, *I*_*N*_, and co-colonized hosts *I*_*VV*_, *I*_*NN*_, and *I*_*VN*_ with vaccine serotypes, non-vaccine serotypes or a combination of the two, respectively. Notice that *S* + ∑ *I* = 1. Upon exposure, a susceptible host can become a single carrier of a VT or NVT pneumococcal serotype. The forces of infection (FOI) are: *λ*_*V*_(*t*) = *β*(*I*_*V*_ + *I*_*VV*_ + *I*_*VN*_/2) for VT, and *λ*_*N*_(*t*) = *β*(*I*_*N*_ + *I*_*NN*_ + *I*_*VN*_/2) for NVT. Single and dual carriers contribute equally to the FOI, and hosts carrying two serotypes transmit either with equal probability. *β* is the per-capita transmission rate. Single carriers can acquire an additional serotype at a reduced rate, modified by a relative competition coefficient *k* ∈ (0, 1) between the resident and the challenge serotype. Pneumococcal carriage is cleared at rate *γ*, regardless of whether hosts are single or dual carriers. Susceptibles are recruited at constant rate *μ*, equal to the per-capita departure rate from the given age class. Immune memory to either set of serotypes is assumed negligible in the time-frame of day care attendance, while serotype-specific immunity is relatively minor compared to the pool of circulating serotypes. Homogeneous mixing is assumed among day-care attendees, and transmission dynamics with the outside community are neglected.

### Calibrating the model to serotype prevalences before vaccination

Under this neutral model, prior to any interventions, vaccine and non-vaccine serotypes coexist at neutrally stable endemic equilibria^34^, which satisfy:

2 $$\begin{array}{c}{S}^{\ast }=\frac{\mu +\gamma }{\beta },\quad {I}_{1}^{\ast }={I}_{V}^{\ast }+{I}_{N}^{\ast }=\frac{(\beta -\gamma -\mu )(\gamma +\mu )}{\beta [\gamma +\mu +k(\beta -\gamma -\mu )]},\\ {I}_{2}^{\ast }={I}_{VV}^{\ast }+{I}_{NN}^{\ast }+{I}_{VN}^{\ast }=\frac{{(\beta -\gamma -\mu )}^{2}k}{\beta [\gamma +\mu +k(\beta -\gamma -\mu )]},\end{array}$$

for the proportion of hosts susceptible at equilibrium, singly-colonized with one serotype, and co-colonized with two serotypes, respectively, where *β* > *γ* + *μ*, equivalently *R*_0_ > 1^64^. When considering serotype distribution, eq. (2) show that the overall pathogen prevalence can be flexibly divided between serotypes in many ways. In a multi-type permutable system, there is however one constraint at a global endemic equilibrium: as soon as the prevalence of one serotype group in single carriage is fixed, (e.g. VT) all other equilibrium prevalences follow as direct functions of it, enabling correlated hierarchies. Thus, although serotypes behave broadly independently, they are coupled through competition for available hosts. Under this model, if the pre-vaccination data are assumed to reflect stationarity, one can infer competition and transmission parameters by fitting endemic prevalence and relative prevalences of single and double carriage. Using *R*_0_ = *β*/(*γ* + *μ*), and ${S}^{\ast }=\mathrm{1/}{R}_{0},{I}_{2}^{\ast }=k({R}_{0}-\mathrm{1)}{I}_{1}^{^{\prime} }\,{\rm{and}}\,{I}_{1}^{\ast }+{I}_{2}^{\ast }=1-\mathrm{1/}{R}_{0}$, at the theoretical endemic equilibrium, the most likely estimates for basic reproduction number and serotype interaction parameter *k* can be obtained from data as:

3 $${\hat{R}}_{0}=\frac{1}{{S}^{\ast }},\quad \hat{k}=\frac{{I}_{2}^{\ast }}{{I}_{1}^{\ast }}\frac{1}{\frac{1}{{S}^{\ast }}-1}\mathrm{.}$$

If, besides overall carriage, the relative prevalence of one serotype group is reported at stationarity, e.g. for VT, ${P}_{V}^{\ast }={I}_{V}^{\ast }+{I}_{VV}^{\ast }+{I}_{NV}^{\ast }\mathrm{/2}$, one can use the above equations in another way, namely to infer the expected proportions of hosts in all other epidemiological classes. In particular, plugging-in the ‘observed’ *P*_*V*_, and a known competition parameter *k* one gets:

4 $${I}_{V}^{\ast }=\frac{{P}_{V}^{\ast }}{1+k({R}_{0}-\mathrm{1)}}\mathrm{.}$$

The other prevalences ${I}_{VV}^{\ast },\,{I}_{N}^{\ast },\,{I}_{NN}^{\ast }$ and ${I}_{VN}^{\ast }$ follow from ${I}_{V}^{\ast }$ iteratively:

5 $${I}_{N}^{\ast }=\frac{{R}_{0}[1-{I}_{V}^{\ast }\mathrm{(1}+k({R}_{0}-\mathrm{1))}]}{{R}_{0}[1+k({R}_{0}-\mathrm{1)}]}$$

6 $${I}_{VV}^{\ast }={({I}_{V}^{\ast })}^{2}k{R}_{0}[1+k({R}_{0}-\mathrm{1)}]$$

7 $${I}_{NN}^{\ast }={({I}_{N}^{\ast })}^{2}k{R}_{0}[1+k({R}_{0}-\mathrm{1)}]$$

8 $${I}_{VN}^{\ast }=1-\frac{1}{{R}_{0}}-({I}_{V}^{\ast }+{I}_{N}^{\ast }+{I}_{VV}^{\ast }+{I}_{NN}^{\ast })\mathrm{.}$$

From a practical point of view, eqs (4–8) represent an analytical trick that can be used to inform *full* initial conditions of a transmission model, in those cases where explicit co-colonization data may be unobserved. In fact, in most datasets considered in this study, except for Norway and Portugal, co-colonization data were not quantified in detail, or not at all. To account for the possibility of underlying co-colonization, and to appropriately initialize model variables in these cases, I treated the pre-vaccination prevalences in France, Hungary, Hong-Kong and Greece, as if they were the endemic prevalences under the above constraints, starting with *P*_*V*_ and fixing *k*, and subsequently unfolding all epidemiological variables from *P*_*V*_ as delineated above.

### Dynamic model with vaccination

In the presence of a vaccination programme, the number of model compartments doubles to 12, but the dynamical equations preserve their basic structure. A fraction *ρ* of all susceptibles is vaccinated at birth. The relative reduction in susceptibility to VT of vaccinated hosts (superscript 1) is given by *w* = 1 − *VE* ∈ (0, 1), compared to those non-vaccinated. It is assumed that vaccine protection acts both at primary and secondary acquisition of a vaccine serotype. The equations are similar for the non-vaccinated hosts (superscript 0), who replenish the susceptible pool, *S* ^0^, at rate *μ*(1 − *ρ*) and experience no protection against pneumococcus. The FOIs are now given by: ${\lambda }_{V}(t)=\beta ({I}_{V}^{1}+{I}_{VV}^{1}+{I}_{VN}^{1}\mathrm{/2}+{I}_{V}^{0}+{I}_{VV}^{0}+{I}_{VN}^{0}\mathrm{/2)}$ for VT, and similarly ${\lambda }_{N}(t)=\beta ({I}_{N}^{1}+{I}_{NN}^{1}+{I}_{VN}^{1}\mathrm{/2}+{I}_{N}^{0}+{I}_{NN}^{0}+{I}_{VN}^{0}\mathrm{/2)}$, for NVT. We still have *S* ^0^ + ∑ *I* ^0^ + *S* ^1^ + ∑ *I* ^1^ = 1. Full model equations are given in Supplementary material S1.

### Linking the model to data: statistical inference

This model is fitted to the cross-sectional prevalence data for children attending day care reported in 6 different countries (Table 2). In cases where full resolution of single and double carriage is available (Portugal and Norway), the explicit pre-vaccine data are directly input as initial conditions of the model, which is subsequently run for each parameter combination. In particular, for Portugal we have for 2001 the following epidemiological observations: [97 77 66 7 5 18] for [*n*_*S*_, *x*_*V*_, *x*_*N*_, *x*_*VV*_, *x*_*NN*_, *x*_*VN*_] (numbers of children in each class), and for 2007: [70 13 125 0 10 2] among vaccinated children, and [60 21 117 1 17 13] among non-vaccinated ones. For Norway, we have the following epidemiological resolution for 2006: [136, 188, 226, 9, 17, 30] for [*n*_*S*_, *x*_*V*_, *x*_*N*_, *x*_*VV*_, *x*_*NN*_, *x*_*VN*_], and for 2008: [118, 82, 328, 2, 36, 26] at the aggregated level over all children in the study of each year. In these two cases, *θ* = (*R*_0_, *k*, *w*) can be estimated accounting for information across all years of the survey, without needing the stationarity assumption for pre-vaccine observations.

In epidemiological studies where only overall prevalence and VT prevalence are reported, the pre-vaccine data are interpreted as a global equilibrium in the absence of intervention, and then, using Eqs (4–8), I derive explicitly initial conditions relative to all classes required by the model, for each value of *R*_0_. In these cases, joint estimation of *θ* = (*R*_0_, *w*) is performed, but *R*_0_ is more tightly coupled to the initial observations pre-vaccination. The equations are solved numerically for different times of follow-up in each study. The dynamic variables *S* ^1^(*t*), ${I}_{V}^{1}(t)$, …, *S* ^0^(*t*), ${I}_{V}^{0}(t)$ are then compared to the epidemiological observations post-PCV7. When only data on prevalence of vaccine serotypes are available: *n*_*V*_ = *x*_*V*_ + *x*_*VV*_ + *x*_*VN*_/2 and *n*_*N*_ = *x*_*N*_ + *x*_*NN*_ + *x*_*VN*_/2, model predictions for ${P}_{V}(t)={I}_{V}^{1}+{I}_{VV}^{1}+{I}_{VN}^{1}\mathrm{/2}+{I}_{V}^{0}+{I}_{VV}^{0}+{I}_{VN}^{0}\mathrm{/2}$ are used for the fitting, instead of all 6 epidemiological variables. Accounting for vaccination coverage and sample sizes across studies, parameter inference is performed using a multinomial likelihood under a Bayesian framework^34^. The likelihood of the data (France, Greece, Hungary, Hong-Kong) is given by:

9 $$L({n}_{S},{n}_{V},{n}_{N}|\theta )=\prod _{{t}_{i}}(\frac{n!}{{n}_{S}!{n}_{V}!{n}_{N}!}{S}^{{n}_{s}}{P}_{V}^{{n}_{V}}{P}_{N}^{{n}_{N}})({t}_{i})$$

where the model variables satisfy: *S*(*t*) + *P*_*V*_(*t*) + *P*_*N*_(*t*) = 1 and the observed data satisfy: *n*_*S*_(*t*) + *n*_*V*_(*t*) + *n*_*N*_(*t*) = *n*(*t*). The expression is similar for Portugal and Norway, but we account for the finer epidemiological resolution of the host population afforded by the data. We used uniform priors in [1, 6] and [0, 1] for *R*_0_ and *w*/*k* respectively. As all children in the studies were day care attendees, we assumed a monthly clearance rate *γ* equal to 0.7. This corresponds to a mean duration of a carriage episode of about 5.7 weeks, as previously documented for this age group^38^. The deterministic model skeleton is used to obtain temporal trajectories for expected prevalence of each carriage type among day-care attendees, which are then integrated within the sampling process, as done in an earlier study^34^. Concerning the entry rate *μ*, I considered the mean duration of DCC attendance per child per setting (Table 1), accounting for the proportions in different age groups and the maximal age of daycare attendees. This resulted in these values of *μ* = 1/*durationDCC* across settings: *μ* =: 0.042 (Portugal), 0.031 (Norway), 0.058 (France), 0.037 (Greece), 0.055 (Hungary) and 0.042 (Hong Kong). The results for model-fitting under a different assumption for *μ* (1/mean age), confirmed similar values for vaccine efficacy, preserving in general the ranking between settings and are detailed in Table S2 and Fig. S4.

The initial conditions for the post-vaccination dynamics are split between vaccinated and non-vaccinated children in the proportions *ρ* and 1 − *ρ*, for initial coverage. When a different coverage is known for each year of follow-up (Table 1), the system is numerically integrated stepwise from year to year, using a different *ρ*, and initializing the system for the next round of integration at the solution obtained up to the last time period. Parameter estimation was performed using the mcmcstat package^63^ in Matlab. Convergence of two independent MCMC chains to the stationary posterior distributions was assessed using the Gelman-Rubin statistic^100^.

## Electronic supplementary material

Supplementary material
