# Supplementary material for: Geographic variation in pneumococcal vaccine efficacy estimated from dynamic modeling of epidemiological data post-PCV7
Source: Sci Rep. 2017 Jun 12;7:3049. doi: 10.1038/s41598-017-02955-y (PMC5468270; doi:10.1038/s41598-017-02955-y)
Supplement: Supplementary file 1 — Supplementary material [file 41598_2017_2955_MOESM1_ESM.pdf]

Supplementary information
Geographic variation in pneumococcal vaccine efficacy estimated from dynamic modeling of epidemiological data post-PCV7
(SREP-16-24050)

Erida Gjini

April 21, 2017

Contents

S1 Pneumococcus colonization model with vaccination **2**

S2 Supporting data **3**
S2.1 Details on serotype prevalences . . . 3
S2.2 Age distribution of children in the different studies . . . 3

S3 Parameter estimates with recruitment rate μ based on mean age **5**

S4 Sensitivity of VE estimates with respect to μ and ρ **6**
S4.1 Keeping step-wise increasing vaccination coverage and varying μ . . . 6
S4.2 Assuming fixed constant coverage throughout the survey . . . 7

S5 Sensitivity of post-vaccine dynamics to R_0 **9**

S6 Accounting for sampling uncertainty in the credible envelopes of temporal trajectories **10**

S7 Variability in environmental and socio-demographic factors across settings **11**

S8 Non-vaccine type similarity across sites **13**

S1 Pneumococcus colonization model with vaccination

The proportions of children in different epidemiological classes change according to the equations:

$$\begin{cases} \text{Vaccinated hosts} \\ \frac{dS^1}{dt} = \mu\rho - S^1(w\lambda_V + \lambda_N) + \gamma I^1_{tot} - \mu S^1 \\ \frac{dI^1_V}{dt} = S^1 w\lambda_V - I^1_V k(w\lambda_V + \lambda_N) - (\gamma+\mu)I^1_V \\ \frac{dI^1_N}{dt} = S^1\lambda_N - I^1_N k(w\lambda_V + \lambda_N) - (\gamma+\mu)I^1_N \\ \frac{dI^1_{VV}}{dt} = I^1_V kw\lambda_V - (\gamma+\mu)I^1_{VV} \\ \frac{dI^1_{NN}}{dt} = I^1_N k\lambda_N - (\gamma+\mu)I^1_{NN} \\ \frac{dI^1_{VN}}{dt} = k\lambda_N I^1_V + kw\lambda_V I^1_N - (\gamma+\mu)I^1_{VN} \end{cases} \qquad \begin{cases} \text{Non-vaccinated hosts} \\ \frac{dS^0}{dt} = \mu(1-\rho) - S^0(\lambda_V + \lambda_N) + \gamma I^0_{tot} - \mu S^0 \\ \frac{dI^0_V}{dt} = S^0\lambda_V - I^0_V k(\lambda_V + \lambda_N) - (\gamma+\mu)I^0_V \\ \frac{dI^0_N}{dt} = S^0\lambda_N - I^0_N k(\lambda_N + \lambda_V) - (\gamma+\mu)I^0_N \\ \frac{dI^0_{VV}}{dt} = I^0_V k\lambda_V - (\gamma+\mu)I^0_{VV} \\ \frac{dI^0_{NN}}{dt} = I^1_N k\lambda_N - (\gamma+\mu)I^0_{NN} \\ \frac{dI^0_{VN}}{dt} = k\lambda_N I^0_V + k\lambda_V I^0_N - (\gamma+\mu)I^0_{VN} \end{cases}$$

where the forces of infection are: $\lambda_V = \beta(I^1_V + I^1_{VV} + I^1_{VN}/2 + I^0_V + I^0_{VV} + I^0_{VN}/2)$; for vaccine serotypes and $\lambda_N = \beta(I^1_N + I^1_{NN} + I^1_{VN}/2 + I^0_N + I^0_{NN} + I^0_{VN}/2)$, for non-vaccine serotypes. The superscripts 0 and 1 denote non-vaccinated and vaccinated children respectively, assumed to homogeneously mix in a day-care setting. The serotype groups share similar transmission, clearance and competition rates.

This model can be reduced to a 10-dimensional system using

$$S^1+I^1_V+I^1_N+I^1_{VV}+I^1_{NN}+I^1_{VN} = S^1+I^1_{tot} = \rho \quad \text{and} \quad S^0+I^0_V+I^0_N+I^0_{VV}+I^0_{NN}+I^0_{VN} = S^0+I^0_{tot} = 1-\rho.$$

Here $\rho \in [0,1]$ denotes the proportion of susceptibles vaccinated at birth, and $w = 1 - VE \in (0,1)$ denotes the reduced rate of acquisition of vaccine serotypes (VT) in vaccinated children.

S2 Supporting data

S2.1 Details on serotype prevalences

Countries	Pre-PCV7				Post-PCV7 (only within NVT)			
**Portugal**	19F<br>14%	23F<br>13%	11A/D<br>11%	6B<br>8%	6A<br>12%	19A<br>10%	6C<br>7%	21<br>6%
**Norway**	6B<br>12%	19F<br>11%	15B/C<br>10.5%	23F<br>10%	15B/C<br>14%	35F<br>7%	35B<br>5%	6C<br>4.5%
**France**	23F<br>30%	6B<br>20%	14<br>15%	19F<br>12%	15<br>20%	19A<br>15%	23A/B<br>10%	6A<br>9%
**Greece**	VT except 19F<br>25%	19F<br>10%	6A<br>11.5%	11A<br>6%	11A<br>1.5%	6A<br>7%	15B<br>5.9%	19A<br>5.7%
**Hungary**	14<br>40%	6A<br>30%	19F<br>10%	other<br>5%	11A<br>31.6%	3<br>7.9%	6A<br>2.6%	31<br>2.6%
**Hong Kong**	6B<br>23%	19F<br>18%	23F<br>13.6%	14<br>8.9%	6A<br>11.8%	6C<br>7.8%	23A<br>5.5%	15B<br>2.6%

Table S1: **Serotype composition before and after PCV7 across countries.** Prevalences are among carriage isolates collected during the first and last sampling time of each survey. Different VT and NVT composition may underlie the variability in the net realized vaccine efficacy across countries.

S2.2 Age distribution of children in the different studies

The expected duration of DCC attendance in each setting, is approximately calculated from the relative frequencies of children in each age sub-group, multiplied by the remaining time until maximum age in that DCC setting.

Age-group	Mean % over all years of survey	Mid-point age	Time remaining in DCC
**Portugal** [i]			
4-11 months	4.2%	7.5 months	64.5 months
12-23 months	7.9%	18 months	54
24-35 months	12.35 %	29.5 months	42.5 months
36-47 months	18.85%	41.5 months	30.5 months
48-60 months	25.2%	54 months	18 months
60-71 months	31.5%	66 months	6 months
Mean		47 months	24 months
Mean reported in paper		47.8 months	
μ		1/47.4=0.021	1/24=0.042
**Norway**			
12-24 months	16.65%	18 months	56 months
24-35 months	19 %	24.5 months	47.5 months
36-47 months	21.2%	41.5 months	30.5 months
48-50 months	24.9%	49 months	23 months
>=60 months	18.25%	66 months	6 months
Mean		40.7 months	32 months
μ		1/40.7=0.025	1/32=0.031
**France**			
3-12 months	14.5%	7.5months	32.5 months
12-24 months	39%	18 months	22 months
24-40 months	46.5%	32 months	8 months
Mean		22.8 months	17 months
μ		1/22.8=0.044	1/17=0.06
**Greece**			
13-23 months	3.5%	18 months	58 months
24-35 months	14.85 %	29.5 months	46.5 months
36-47 months	33.42%	41.5 months	34.5 months
48-76 months	48.23%	62 months	14 months
Mean calculated here		48.8 months	27.2 months
Mean reported in paper		46.8 months	
μ		1/47=0.021	1/27.2=0.037
**Hungary**			
36-72 months	100%	54 months	18 months
Mean assumed		54 months	18 months
μ		1/54=0.018	1/18=0.055
**Hong-Kong**			
24-72 months	100%	48 months	24 months
Mean assumed		48 months	24 months
Reported in the study		49.2 months	
μ		1/49.2=0.020	1/24=0.042

[i] Mean age=sum([4.2,7.9,12.35,18.85,25.2,31.5].*[7.5,18,29.5,41.5,54,66])/100 ;

Mean DCC time= sum([4.2,7.9,12.35,18.85,25.2,31.5].* [64.5,54,42.5,30.5,18,6])/100;

S3 Parameter estimates with recruitment rate μ based on mean age

Table S2: **Parameter estimates for pneumococcus transmission and PCV7 vaccine efficacy (VE) in day care settings, assuming a different birth/death rate μ=1/mean age in the SIS model.** From Table 1 in the paper we can obtain μ as: 0.021 (Portugal), 0.025 (Norway), 0.043 (France), 0.021 (Greece), 0.018 (Hungary) and 0.021 (Hong Kong). Here, the means of the posterior distributions and the 95% credible intervals are given. The parameter k is set to a fixed value in 3 cases, equal to 0.05. The transmission rate per month is derived as: $\beta(i) = \frac{R_0(i)}{\gamma+\mu(i)}$.

Country	Basic reproduction number R_0	Competition parameter k	Vaccine efficacy VE	Transmission rate β
Portugal	3.20 (2.88, 3.57)	0.08 (0.06,0.11)	0.12 (0.10,0.14)	2.30 (2.07,2.57)
Norway	4.74 (4.00,5.34)	0.04 (0.03, 0.05)	0.44 (0.36, 0.52)	3.44 (2.90, 3.87)
France	2.22 (2.14, 2.31)	-	0.21 (0.18, 0.24)	1.65 (1.59,1.72)
Greece	1.85 (1.79, 1.91)	0.045 (0.04,0.05)	0.25 (0.20, 0.29)	1.34 (1.29,1.38)
Hungary	1.55 (1.49, 1.62)	-	0.46 (0.35, 0.58)	1.11 (1.07,1.16)
Hong Kong	1.23 (1.22, 1.24)	-	0.12 (0.08, 0.16)	0.88 (0.88,0.89)

S4 Sensitivity of VE estimates with respect to μ and ρ

S4.1 Keeping step-wise increasing vaccination coverage and varying μ

I performed a sensitivity analysis for each country, varying susceptible recruitment rate μ in the range spanned by 1/mean age and 1/mean DCC attendance, using the same step-wise increasing vaccination coverages (Table 1 in the paper) and visualized here in Figure S1.

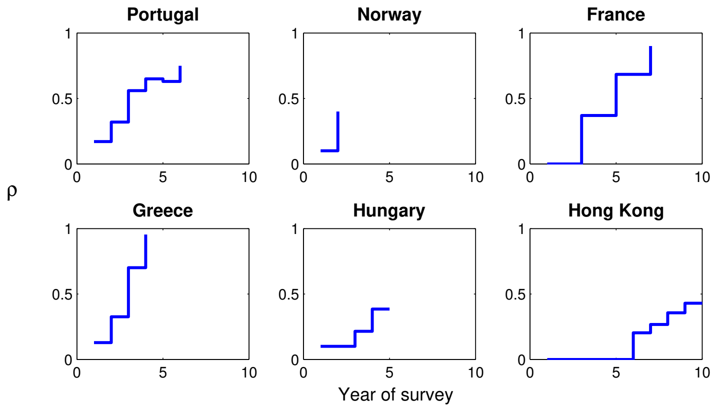

Figure S1: **The increasing vaccine coverage rates in each setting.** As extracted from the papers and reported in Table 1. These correspond to the updated ρ parameter values at the beginning of each simulation period corresponding to one year in the epidemiological dynamics with vaccination.

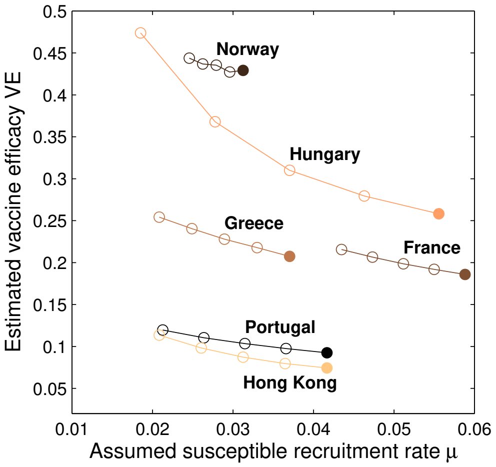

Figure S2: **The relationship between assumed susceptible recruitment rate and estimated vaccine efficacies.** For each country a range of five μ values was tested between 1/mean age and 1/mean DCC attendance. The estimated VE values from model fitting (same MCMC procedure explained in the paper) with step-wise increasing vaccination coverage are denoted with the colored circles, where the right-most one (filled) corresponds to the values reported in the paper.

The values of estimated vaccine efficacies are robust to changes in μ, as shown in Figure S2, and importantly, the ranking between countries is preserved. We observe that as the assumed μ increases, the estimate of VE decreases slightly for the same datasets under the original vaccination coverages. Yet, the change is not major, except for Hungary, where we cover the largest uncertainty in μ values. Furthermore the ranking is still preserved between Norway and Hungary displaying higher realized VE, than France and Greece, with Portugal and Hong-Kong displaying lowest realized protection, independently of μ.

S4.2 Assuming fixed constant coverage throughout the survey

Next, I performed the same sensitivity analysis using a continuous vaccination assumption instead (as opposed to a step-wise increasing coverage), holding fixed the vaccination coverage per year, throughout the years of the survey. This is a drastic qualitative change in those model parameters which are assumed known. Even though it may be unrealistic, it is presented to complete the overall picture and clarify the importance of vaccination coverage. The range of variation for the constant ρ in this second sensitivity analysis was chosen relative to the median of the values in Table 1 of the paper, set typically from $0.5 \times median$ to $1.5 \times median$, in each country. The medians are given in table S3. The results of model fitting under this assumption are shown in Figures S3-S4.

Table S3: **Median and mean vaccination coverage in different countries, calculated over the entire survey period (as in Table 1 in the paper).** These values were used to fit a different version of the model with constant coverage throughout the study period, starting from $t = 0$. This is qualitatively different, and somewhat an over-simplification, compared to the increasing step-wise coverage, adopted in the paper, which likely represents more accurately the actual pace of the intervention. However these values are used in the sensitivity analysis, presented for completion.

Country	Median ρ	Mean ρ
Portugal	0.59	0.51
Norway	0.25	0.25
France	0.37	0.43
Greece	0.51	0.53
Hungary	0.21	0.24
Hong Kong	0.10	0.17

The values for estimated VE in these cases, assuming a fixed ρ throughout the survey period, are generally lower, in the range of 8-15% for Hong-Kong, Portugal, Greece, France and Hungary, and about 24% for Norway this time. These values are about half the values obtained with the step-wise increasing $\rho(t)$. When $\rho(t)$ changes over time, as assumed in the paper, low coverage values in the beginning of an intervention, increase VE parameter estimates for the same observations, an effect that is stronger than high vaccination coverage later on: early on processes feed back more strongly in the overall nonlinear dynamics of the system. That is why, for example, the VE estimate for Norway is about 40%, under the stepwise coverage assumption, and appears as 24% in the constant coverage model. From this analysis, we can conclude that the order-ranking of site-specific VE estimates is generally preserved, independently of slight changes in assumptions on μ and ρ. Clearly, a more realistic description of vaccination implementation in each country must take into account changes from year to year, thus the step-wise vaccination model, as adopted in the paper (and extended in S6.1), remains a more appropriate approximation, likely yielding better parameter accuracy.

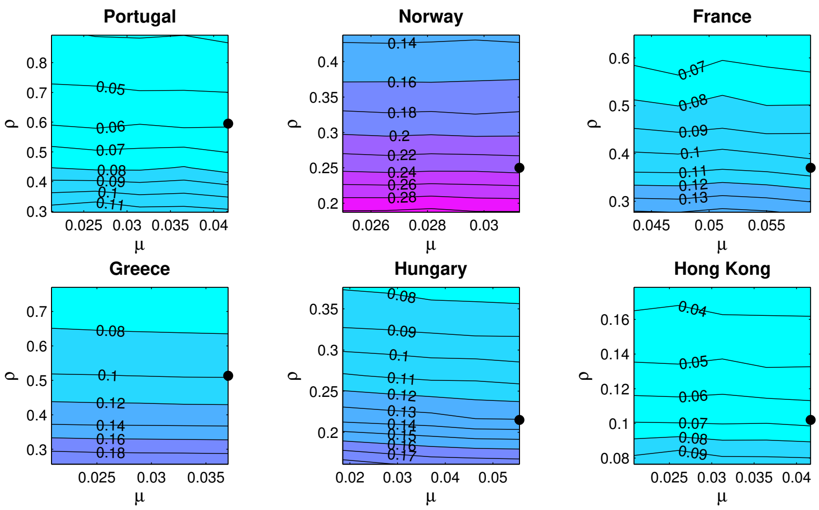

Figure S3: **Sensitivity analysis for estimated VE assuming a range of susceptible recruitment rates and a range of vaccination coverage held constant throughout the survey period.** The range of vaccination coverages ρ varied around the median of the values reported in Table 1. The range of μ varied between 1/mean age and 1/mean DCC attendance. Model-fitting using the same MCMC procedure was performed on the data for each combination of μ and ρ, and the resulting VE estimates are shown as a contour plot. The filled circle reflects the value of VE corresponding to $\mu = 1/meanDCC$ assumed in the paper, and $\rho = median$ of the values in Table 1.

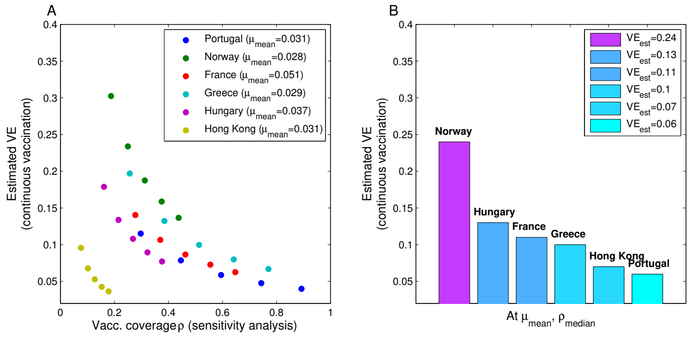

Figure S4: **Summary of the relationship between assumed vaccination coverage and estimated vaccine efficacies, under a constant coverage model.** This figure summarizes figure S3. For each combination (ρ, μ), setting-specific estimates of VE were obtained, then averaged over μ. A) VE averages over μ for five values of vaccination coverage (colored circles). The higher the fixed coverage assumed, the lower the vaccine efficacy estimated, to explain the same dataset. B) The VE estimates assuming $\mu = \mu_{mean}$ and $\rho = median$ fixed for each country over the survey period. The ranking between settings is preserved, although the mean efficacy appears lower. The bias in estimates toward lower values is due to the reduction from a step-wise increasing coverage to a constant coverage assumption, which is an over-simplification.

S5 Sensitivity of post-vaccine dynamics to R_0

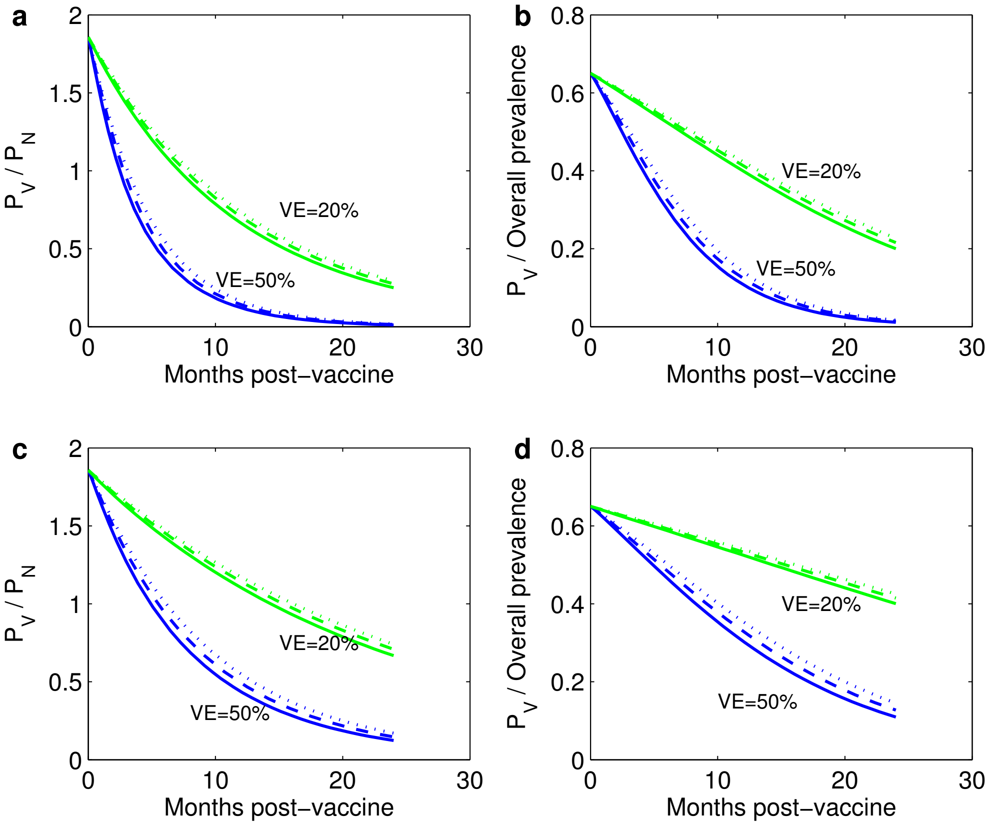

Figure S5: **The relationship between target serotype decline after vaccination and transmission intensity in a neutral model.** a-b) Vaccine coverage $\rho = 0.5$. c-d) Vaccine coverage $\rho = 0.25$. Panels show prevalence ratio P_V/P_N of target vs. non-target serotype prevalence against time, and relative prevalence $P_V/(P_V + P_N)$ of target serotypes following vaccination for the same initial conditions, for two different vaccine efficacies (green and blue lines), and R_0. The line styles depict different values of R_0: 5 (solid line), 2.5 (dashed), 1.5 (dotted). Other parameters: competition coefficient $k = 0.05$; monthly clearance rate $\gamma = 0.7$; monthly susceptible recruitment rate $\mu = 0.02$.

S6 Accounting for sampling uncertainty in the credible envelopes of temporal trajectories

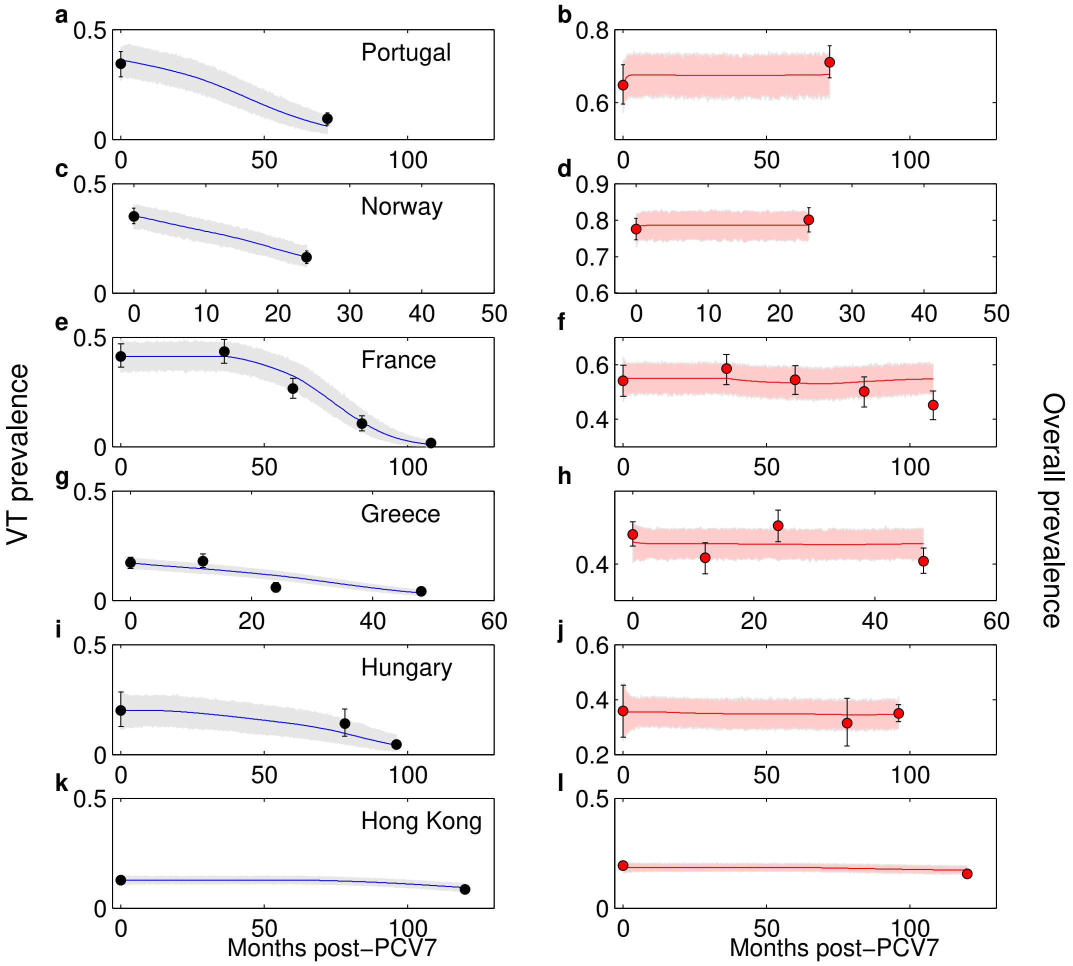

Figure S6: **95% credible envelopes for model trajectories, accounting for sampling process over time.** These envelopes capture simultaneously uncertainty around the posterior parameter values, and the variability introduced by the sampling process. Here the same sample size was assumed in each setting equal to the mean in Table 1, fixed throughout the years of the survey. 500 model simulations with random parameter combinations, and superimposed sampling of binomial proportions were considered to compute the 95% CI of model-predicted dynamics.

S7 Variability in environmental and socio-demographic factors across settings

Table S4: **Temperature estimates for geographical sites.** Retrieved last in January 2017. Mean daily temperatures, averaged over the year in each setting are used for the regression analysis in Figure 5 of the paper.

Country	Value (degrees Celsius)	Reference
Oeiras, (Lisbon) Portugal	17.5	(1)
Oslo, Norway	5.69	(2)
Alpes Maritimes, France	16	(3)
Larissa, Greece	15.7	(4)
Szeged, Hungary	10.45	(5)
Hong Kong, China	24.1	(6)

Table S5: **Gini coefficients for different countries.** Retrieved last in January 2017. Means were computed between the values in the first and last year of each survey when available.

Country	Gini index	Reference
Portugal	38	(7)
Norway	27.2	(8)
France	31.5	(9)
Greece	34.6	(10)
Hungary	26.9	(11)
Hong Kong	47.5	(12)

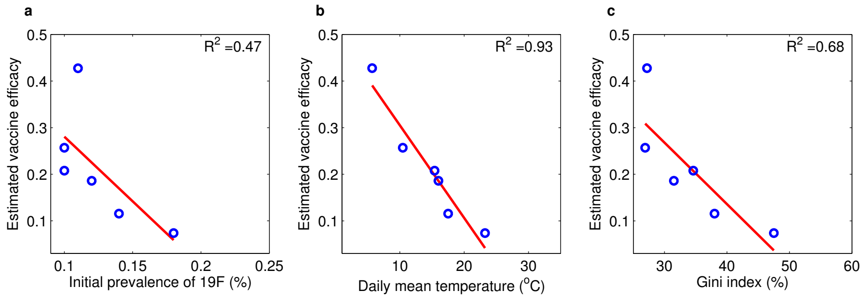

Figure S7: **A second version of figure 5 in the paper with fitted regression lines.**

References

[1] http://www.ipma.pt/pt/oclima/normais.clima/1981-2010/012/. Retrieved 2017-01-05.

[2] https://met.no/Klima/Klimastatistikk/Vanlig_var/Sor-_og_Austlandet/Blindern_Oslo/. Retrieved 2017-01-05.

[3] http://www.lameteo.org/index.php/climatologie/1543-normales-climatiques-1981-2010-nice. Retrieved 2017-01-05.

[4] ftp://ftp.atdd.noaa.gov/pub/GCOS/WMO-Normals/TABLES/REG_VI/GR/16648.TXT. Retrieved 2017-01-05.

[5] http://www.hko.gov.hk/wxinfo/climat/world/eng/europe/ger_pl/szeged_e.htm. Retrieved 2017-01-05.

[6] http://www.weather.gov.hk/aviat/amt_e/monthlymean_e.htm. Retrieved 2017-01-05.

[7] http://data.worldbank.org/indicator/SI.POV.GINI?end=2007&locations=PT&start=2001. Retrieved 2017-01-05.

[8] http://data.worldbank.org/indicator/SI.POV.GINI?end=2008&locations=NO&start=2006. Retrieved 2017-01-05.

[9] http://data.worldbank.org/indicator/SI.POV.GINI?end=2008&locations=FR&start=1999. Retrieved 2017-01-05.

[10] http://data.worldbank.org/indicator/SI.POV.GINI?end=2009&locations=GR&start=2005. Retrieved 2017-01-05.

[11] http://data.worldbank.org/indicator/SI.POV.GINI?end=2009&locations=HU&start=1999. Retrieved 2017-01-05.

[12] http://www.hkeconomy.gov.hk/en/pdf/gini_comparison.pdf. Retrieved 2017-01-05.

S8 Non-vaccine type similarity across sites

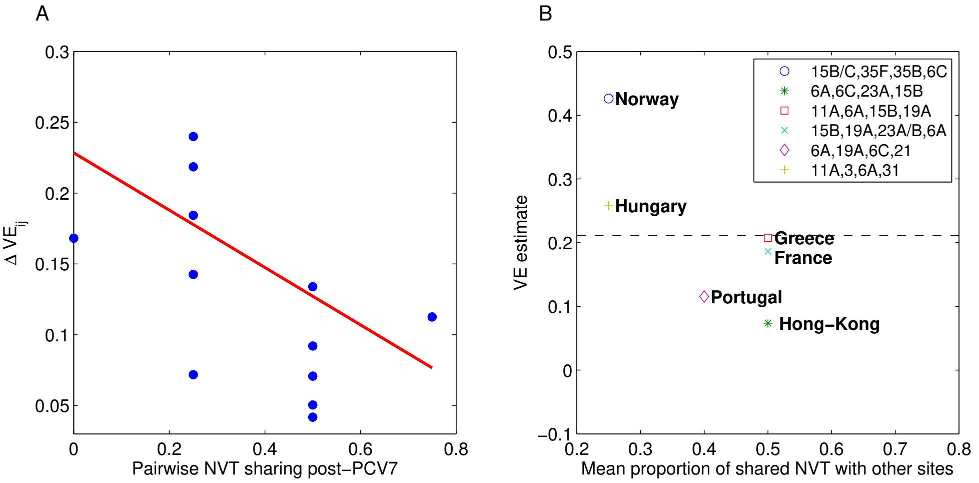

Figure S8: **Similarity in non-vaccine type composition and similarity of model-based vaccine efficacy estimates.** A) Pairwise comparison between settings (Table S1) with regards to proportion of shared NVTs among the 4 top-most prevalent post-PCV7 (number/4). Assuming independence between pairwise comparisons, the association emerges to be significant (p-value for slope $p < 0.05$). B) Averaged NVT sharing across pairs for each setting (mean over 5 pair-wise comparisons) and deviation from mean VE (dashed line). A decreasing trend, although it must be taken with caution given the limitations of the present dataset, suggests that ecological niche overlap, even though too coarsely captured via NVT subset overlap, may be yet another factor influencing site-specific variation in realized vaccine protection against PCV7 pneumococcal serotypes.
